# Evolutionary and differential psychology: conceptual conflicts and the path to integration

**DOI:** 10.3389/fpsyg.2013.00655

**Published:** 2013-09-23

**Authors:** Tim Marsh, Simon Boag

**Affiliations:** Department of Psychology, Macquarie UniversitySydney, NSW, Australia

**Keywords:** bottom-up explanation, differential psychology, evolutionary psychology, individual differences, integration, top-down explanation, unification of psychology

## Abstract

Evolutionary psychology has seen the majority of its success exploring adaptive features of the mind believed to be ubiquitous across our species. This has given rise to the belief that the adaptationist approach has little to offer the field of differential psychology, which concerns itself exclusively with the ways in which individuals systematically differ. By framing the historical origins of both disciplines, and exploring the means through which they each address the unique challenges of psychological description and explanation, the present article identifies the conceptual and theoretical problems that have kept differential psychology isolated not only from evolutionary psychology, but from explanatory approaches in general. Paying special attention to these conceptual problems, the authors review how these difficulties are being overcome by contemporary evolutionary research, and offer instructive suggestions concerning how differential researchers (and others) can best build upon these innovations.

Psychology has been described as a science impaired by disunity (Gladin, 1961; Meehl, 1978; Kantor, 1979; Staats, 1983, 1999; de Groot, 1990; Yanchar and Slife, 1997; Henriques, 2003, 2004, 2011; Goertzen, 2008, 2010; Mandler, 2011). There is, however, disagreement over precisely how large a problem theoretical and institutional disunity is for psychologists and behavioral scientists in general (Dixon, 1983; Baars, 1984, 1985; Matarazzo, 1987, 1992; Bower, 1993; Neisser, 1995; Kelly, 1998; Kassinove, 2002; Stam, 2004). Nevertheless, integration is widely considered a desirable course of action, if only for the potential benefits of combining disparate theories and findings within a common conceptual space (Staats, 1999; Henriques, 2003, 2011; Goertzen, 2008). In recent years, the *adaptationist* approach of evolutionary psychology has emerged as strong candidate for central inclusion in a unifying meta-theory of psychology (Penke et al., 2007a,b; Tooby and Cosmides, 2007; Webster, 2007; Daly and Wilson, 2008; Buss, 2009). Stated briefly, adaptationism is a paradigm for analyzing the physical and behavioral characteristics of organisms by focusing on functionally complex features which can only arise through natural selective pressures (see Daly and Wilson, 1999 for a brief review of the origins of adaptationism in behavioral science). Despite some enduring camps of resistance (Rose and Rose, 2000; Buller, 2005; Richardson, 2007), the literature shows a trend of increasing acceptance of adaptationism in diverse fields of psychology (Confer et al., 2010; Fitzgerald and Whitaker, 2010). Many recent unification efforts orient around evolutionary theories and approaches (Sternberg and Grigorenko, 2001; Henriques, 2003, 2004, 2008, 2011; Gintis, 2007). Nevertheless, although the adaptationist approach can and has been readily applied to an extensive range of psychological phenomena, as highlighted in Confer et al. (2010) some areas of psychology pose unique theoretical and methodological difficulties, which evolutionists must (befittingly) adapt to. Perhaps the largest category of phenomena that demands a revision of traditional adaptationist analyses is the systematic occurrence of variation in normative psychological characteristics, the domain of personality and individual differences (Buss, 2009; Buss and Hawley, 2011). As Confer et al. (2010) summaries: “Evolutionary psychology has been far more successful in predicting and explaining species-typical and sex-differentiated psychological adaptations than explaining variation within species or within the sexes” (p. 123). The recent innovations on this front discussed later in this article are best appreciated relative to the history and present state of traditional differential psychology.

Over the past century, the study of normative individual differences in thought, behavior and ability (hereafter referred to by the umbrella term “differential psychology”) has become one of the largest and most popular arms of psychological science (Lubinski, 2000; Borghans et al., 2011). Differential psychology has intimate ties to multiple fields of applied psychology, including psychometric assessment, developmental and educational psychology, lifestyle and vocational adjustment, and our shifting conceptions of psychopathology (Lubinski, 2000). Despite this, differential psychology has a long history of remaining largely theoretically autonomous from related sub-disciplines of psychology. To this day, there is little cross-pollination between even the largest areas of differential psychology and their immediately adjacent research fields (Mischel, 1968, 1973; Cervone, 1991; Borsboom, 2005; Cramer et al., 2010). To illustrate the point, the differential psychology domain of cognitive ability/intelligence testing has developed largely independent of the insights of functional cognitive psychology (Cronbach, 1957; Neisser et al., 1996; Garlick, 2002, 2003; Anderson, 2005). Additionally, differential trait theories have become a prevailing approach in the study of personality, whilst remaining predominantly separate from other leading conceptions and models within personality psychology (see Block, 1989, contrasted with 2010, for examples both before and after the rise of the Five Factor Approach). Evolutionary psychology represents only the most recent theoretical approach that must now struggle to integrate with the relatively independent niche carved out by the traditions of differential psychology.

While both evolutionary psychology and differential psychology are immensely diverse and heterogeneous fields, the arguments of this paper seek to cast as wide and as relevant a net as possible. As such, primary focus shall be given to fundamental conceptual and methodological elements that are near-ubiquitous characteristics of the respective fields, with more specific examples drawn from the most relevant and representative research areas available. By utilizing some often overlooked distinctions from the wider philosophy of science, examining the fundamental scientific tasks of *description* and *explanation* (and beyond this, *forms* of explanation), the authors seek to explore the apparent theoretical isolation of differential psychology, and argue that integration is possible only when descriptive efforts are designed to inform causal explanations. In approaching this contentious topic from a neglected theoretical perspective, this paper contributes a new argument to the collective evolutionary-differential integration efforts started by David Buss almost 30 years ago (1984), an argument designed to address the fundamental conceptual concerns echoed by some critics of evolutionary psychology (Buller, 2005; Richardson, 2007). The current state of integration efforts and possible future avenues for individual differences research will also be discussed.

## A common ancestor

During the formative period of the late 1800s, the precursors of both evolutionary and differential psychology were initially proposed as means to a common end. Methodologies emphasizing species-typical features and those emphasizing between-subjects variation share a number of common ancestors, perhaps the most illustrative of which is the career of Sir Francis Galton (Galton, 1889; Allen, 2002). Whereas vaguely evolutionarily-guided biological insights have shaped such influential theories as those of Sigmund Freud (Young, 2006) and B. F. Skinner (Skinner, 1966, 1984), Galton (a half-cousin to Charles Darwin) focused very specifically on the application of several Darwinian principles to studying the human species (Forest, 1995).

Galton is of central relevance to the history of personality and individual differences (Bynum, 2002), having pioneered the psychometric assessment of both abilities and dispositions, first articulating the paradigm of “nature vs. nurture,” and developing statistical methods oriented around correlation and the use of regression toward the mean with standard deviations (Simonton, 2003). Though now remembered poorly for his advocacy of eugenics, Galton's endeavors in measuring variability and supplementing selective pressures in human populations were two necessary components of a single ambition: to preserve and aid the evolution of the human species, with particular regard towards human intelligence and character (Jensen, 2002; Seligman, 2002).

Galton understood that the most crucial aspects of Darwin's theory of evolution by natural selection can be broken down into two discrete concepts. Firstly, that all populations of organisms contain some meaningfully heritable variation, and second, that the differential efficacy of these variants with regard to the demands of survival and reproduction produce a form of selection (Darwin, 1859, 1871). The representative properties of any species will reliably change over time, in such a manner as to increase their contextual reproductive success, so long as sufficient variation and selection can occur. In the introductory pages of their recent edited book, Buss and Hawley (2011) state flatly: “Individual differences are indispensible for natural selection. Without heritable variants, natural selection—the only known process capable of creating and maintaining functional adaptations—could not occur.” (p. ix).

From this perspective, we can appreciate, in much the same manner as Galton and his contemporaries, that the study of population variation and the study of selective pressures are two sides to the same coin. Both aspects are necessary to understand the history and present-state of biological and psychological functioning, and our richest insights must be born of complex interactions between the two. Thus, to understand the apparent rift that has since formed between these two philosophically congruent fields, one must turn an eye to their separate trajectories of historical implementation.

## Contrasting focuses and conflicting methods

The technological progression of the past 150 years has precluded the study of human variation and the study of human evolutionary design from developing hand-in-hand. Darwin's original articulation of evolutionary theory was inhibited from its inception by a lack of insight into the molecular mechanisms of heredity. While basic inheritance of biological traits had been well-observed, it was not until more than 50 years later, when Mendel's theories of genetics and Morgan's chromosome theory were integrated, that biologists were in a position to undertake meaningful investigations into the propagation of varying traits throughout a population (Huxley, 1942; Dennett, 1995a; Bowler, 2003; Olsson et al., 2006). From the beginning of the 20th century, the study of selective pressures was impaired for many decades, awaiting both the gradual stockpiling of heritability data, and the development of molecular-genetic and computer-modeling techniques.

During this time, several disciplines focusing on measuring and predicting population variation thrived (Stern, 1911), most notably the burgeoning field of differential psychology (Lamiell, 2003; Bergman and Trost, 2006; Uher, 2008). These early endeavors did not suffer at all in the absence of a study of selection, for the findings themselves were considered simply a cross-section of a presumably changing population. Since selection can only occur between generations, only measures of variation spanning over two or more generations would require insights into selection to be understood. It is during this period, while selection-focused sciences were still handicapped by technology that differential psychology flourished.

The early differential techniques fed strongly into many of the experimental psychology approaches of the era (Tucker et al., 2005), enduring the dominance of behaviorism to then be reinvigorated by the cognitive revolution that followed (Block, 1989; Baum, 1994; Mandler, 2002; Miller, 2003). During this time, differential psychologists distanced themselves from the rapidly shifting theories in related fields, and came to rely heavily on their robust statistical constructs and improving ability to predict outcomes (Lubinski, 2000; Maltby et al., 2007). Growing beyond initial interests in improving the process of military recruitment, differential psychology forged close relationships with many areas of applied psychology (Tyler, 1965). The domains of personality- and intelligence-testing in particular, grew ever-more prominent in predicting and informing outcomes including educational development, vocational selection, risk-management, and mental and physical health outcomes, to name only a few (Karasek, 1979; Lubinski, 2000; Marks et al., 2005; Reisner, 2005; Maltby et al., 2007).

From the late 1980s to the present day, differential psychology has fortified its position as a central pillar of psychological science, with influential constructs such as the “g” factor of intelligence and trait models of personality standing at the center of decades of empirical support (Chamorro-Premuzic and Furhnam, 2006; Reeve and Charles, 2008; Block, 2010). Contemporary personality and individual differences research is defined by constructs that rely little on grounding theories, but rather, are built on robust statistical data drawn from large populations (Borsboom, 2005). One might thus presume that researchers would regard differential psychology constructs as having limited or strictly instrumental use, relative to the explanatory theories they diverge from. To the contrary, however, trends in the literature suggest that differential constructs are thriving, while theory-based and qualitative research approaches are systematically disfavored (Rogers, 2000). One explanation for this bias is the “quantitative imperative” (Michell, 1990, 2003a,b): “The quantitative imperative is the view that in science, when you cannot measure, you do not really know what you're talking about, but when you can, you do” (Michell, 2003a p.5). According to Michell (2005) this quantitative imperative acts both as an explicit principle and as a subtle network of social and institutional biases. Through such influences, the individual differences field has come to embrace its historical overspecialization in nomothetic statistics as evidence of true scientific validity (Borsboom et al., 2004; Borsboom, 2005).

In contrast, early attempts to address human psychological phenomena with reference to selective pressures only began to emerge in the latter half of the 20^th^ century, under the umbrella-term “sociobiology” (Hamilton, 1954; Wilson, 1975). These attempts ultimately proved conceptually inadequate, as many were highly reminiscent of the genetic-determinist theories then-prevalent in ethology and zoology, or depended intimately on the then-controversial prospect of group-selection (Gould and Lewontin, 1979; Gould, 1981; Vining, 1986). Only in the late 1980s did the adaptationist paradigm of evolutionary psychology fully emerge (Buss, 1984, 1995; Cosmides and Tooby, 1989; Tooby and Cosmides, 1989), requiring another decade of development before the approach became widely acknowledged (Confer et al., 2010; Fitzgerald and Whitaker, 2010). Evolutionary psychologists established a refined adaptationist approach, drawing from contemporary cognitive psychology to strongly emphasize the modularity and domain-specificity of hypothesized psychological adaptations (Cosmides and Tooby, 1987, 1997; Nesse and Lloyd, 1992; Pinker and Bloom, 1992; Pinker, 1997). Evolutionary psychology specifically targeted those features of psychological functioning which are species-typical mechanisms that evolved in response to the recurring survival and reproductive challenges of Pleistocene epoch human ancestors (Buss, 1999, 2005). Such species-typical features offer an important means of empirical hypothesis-testing, as only ubiquitous, biologically-based features are likely to exist in similar forms cross-culturally (Buss et al., 1990; Barkow et al., 1992; Tooby and Cosmides, 1992; Buss, 2005).

As of the beginning of the 21st century, an apt summary of the two fields was that evolutionary psychology focuses on the features which are shared across our species, while differential psychology focuses on the ways in which the members of our species systematically differ (Borghans et al., 2011). Given their shared origins, one might presume that findings of the two approaches must be intrinsically disposed to integration. However, despite some attempts dating back to the formative years of evolutionary psychology (Buss, 1984, 1991), integration efforts have faced theoretical and practical difficulties, to a degree that some view as an interdisciplinary hostility (Anderson, 2005; Muncer, 2011).

To understand this divide, it is necessary to explore some of the unique conceptual challenges that afflict psychology research more so than almost any other field of science. These conceptual difficulties lend disproportionate weight to variations in approach and methods, and are a driving force behind the characteristic rifts between the sub-disciplines of psychology (see Goertzen, 2008 for a diverse account). Moreover, an exploration of these issues can offer an insight into the asymmetrical unification attempts between evolutionary and differential psychology especially (Pinker, 2002; Tooby et al., 2005; Rodeheffer et al., 2011), and provide specific means through which such conflicts may, and must, be overcome.

## The unique challenges of psychological inquiry

In order to discuss the challenges that psychology faces as a science, it is necessary to first clarify precisely what is meant by “science.” While views on what constitutes “science” vary (Salmon, 1989; Gaukroger, 2006), the scientific enterprise generally consists of two major elements: the systematic observation and description of a particular set of natural phenomena, and the theory-guided explanation of the causes of said phenomena (Wilson, 1998; Cervone, 1999; Boag, 2011). In employing this definition, the authors seek to approximate the position advocated by Wilson (1998), and emphasize that the role of science is to “factor out human values” through procedural error-checking, with the goal of developing “representations of reality that are as accurate as possible.”

### A science within a black box

To understand the conceptual difficulties of psychological inquiry, it is illustrative to regard all aspects that cannot be immediately observed as existing within a figurative “black box.” In the engineering sciences, a “black box” is the catch-all term for any system that has traceable outputs, and at least somewhat traceable inputs, but of which one can gain little to no direct insight into the internal processes that bridge between them (House, 1991; Nairne, 1997; Astbury and Leeuw, 2010). The black box nature of psychological phenomena poses few difficulties for the tasks of observation and description, as these are generally concerned with the system's inputs and outputs (behaviors, levels of activity, etc.). Black boxes do, however, pose substantial challenges to the task of explanation.

Since a phenomenon can only be explained via reference to those related antecedents which, in the past, caused its current state, black box systems concern observable phenomena (the outputs and inputs of the black box) which have causal relations to elements and/or objects that cannot be observed (Kitcher, 1985; Salmon, 1989; Cervone, 1999; Ketelaar and Ellis, 2000; Hüttemann and Love, 2011). Any explanatory account of the inputs or outputs of a black box must by necessity contain some incomplete space, permitting nothing more concrete than speculation. As an example, consider an alarm clock, with the standard inputs (an electric power cord) and outputs (patterns of light and sound). While one might reasonably presume that the device contains electrical circuits that keep time, we must acknowledge that without opening the box, one can only speculate as to precisely what form these internal components take. Relying only on the inputs and outputs, we have no means with which to distinguish between multiple options that achieve the same overt patterns, for any mechanism capable of keeping time, regardless of method, would be functionally identical.

This limitation underpins one of the defining characteristics of the scientific method: hypothesis-testing, which acts as an algorithmic process comprised of both the generative and selective phases that most diagnostic procedures rely on (Fisher, 1925; Kaplan, 1964). It is common when investigating natural phenomena to only have a subset amenable to direct measurement. As such, hypothesis-testing is employed to interpret predictive patterns in that which is observed, to infer the possible characteristics of the variables that cannot be observed (Bunge, 1963; Beizer, 1995). The black box metaphor need not only refer to physical limitations, but rather, a situation can present as a “black box” relative to the means of the investigator. Any situation is figuratively a black box, if vital explanatory details are amenable only to the hypothesis-testing of peripheral phenomena.

From a methodological perspective, the fundamental limit to the utility of hypothesis-testing is that a theory could only be definitively “proven” via the exhaustive disproving of all possible alternative hypotheses. For most kinds of black box situations, there are an effectively infinite number of alternative hypotheses concerning the character of the hidden sections. Thus, heuristics that guide investigators toward testing the most likely or plausible hypotheses are the saving grace that renders actual hypothesis-testing possible. Such heuristics are generally drawn from theory, however, and the more extensive or multi-layered the black box is, the more potentially inscrutable the input-output contingencies become (Fisher, 1925; Bunge, 1963; Kaplan, 1964; Beizer, 1995; Kaplan and Craver, 2011; also see Cervone, 1999 for psychology-specific discussion).

This fundamental challenge of constructing explanatory theories for complex black box phenomena is the central philosophical and conceptual difficulty that defines psychology as a science. To a degree largely unshared by any other natural science, the black box phenomena comprising the information-processing systems of humans and other animals are near-insurmountably complex. The subject matter of psychology concerns highly interpretable stimuli, passing through immensely long, largely immeasurable, variable and internally-referential causal sequences (Jaszczolt, 1996), to emerge as behavioral outputs that are themselves highly interpretable (De Los Reyes and Kazdin, 2008).

As an immediate consequence of this, sub-disciplines of psychology are particularly vulnerable to sectarianism and disunity. Most fields of psychology have, understandably, built their theories and explanatory models using those insights most conducive to answering their specific research questions (Matarazzo, 1992; Kelly, 1998). As a result many fields of psychology make dissonant or contradictory pragmatic assumptions about those aspects of the mental black box that they are not presently addressing. The mutually incompatible assumptions that characterize different research approaches appear to be responsible for the majority of institutional disunity in psychology, including the rift between evolutionary and differential psychology.

### Refining explanatory theories

Although description is fundamentally necessary for explanation to occur, explanation is arguably the highest goal of science (Wilson, 1998; Cervone, 1999, 2005). As such, a research approach in psychology is perhaps the best judged in terms of its ability to constrain theories and predictions, so as to reliably draw maximum utility out of practical hypothesis-testing (see Kaplan, 1964, chapter 2 for general elaboration; for discussion specific to evolutionary psychology, see Resnik, 1996; Sober, 2000; Lewens, 2002).

There are, in general, three means of informing an explanatory theory prior to (or in conjunction with) prediction-testing of input-output contingencies (Bunge, 1963). The first, and often most difficult, option is to attempt to directly measure the contents of the black box. In psychology, this may be achieved in two ways: directly, through the use of various neuroimaging technologies, and analogously, through the invasive (generally surgical) manipulations of non-human animals. While there is not nearly sufficient space here to discuss the valuable psychological insights that have been gathered through these respective methods (for some key topics, see: Stevenson and Goldworth, 2002; Bennett and Hacker, 2003; Tashiro, 2004; Filler, 2009; Dietrich and Kanso, 2010), for the specific purpose of theory-building their utility is none-the-less akin to that of standard observation-based methods. While many intuitively assume that the real-time outputs of fMRI scans provide privileged access to the content of the mind, neuroimaging technologies only provide us with activity patterns, which while potentially closely correlated with the information-transformations of the mind, do not constitute direct measurement of the phenomena in question (Caplan, 2009). Even if neuroimaging techniques were so technologically refined as to accurately discern specific action potentials and the dynamic dendrite configurations of individual neurons, the interpretation of these patterns into meaningful psychological content could still only be achieved via detailed correlation with some other source of insight into said processes (Bennett and Hacker, 2003). Though immensely instructive, these methods cannot side-step the fundamental difficulties of hypothesis-testing, but rather can only be taken alongside psychological testing as means of refining existing hypotheses (Caplan, 2009; Filler, 2009).

The second option for refining theories independent of testing involves the use of logical inference to determine what must be the necessary minimum requirements of the systems in question, assuming that said systems are physically internally consistent. This method is extensively employed in computational cognitive psychology (Fodor, 1975, 1983), and is the guiding heuristic of all computational models (Neisser, 1967; Boden and Mellor, 1984; see chapter 4 of Boden, 2006 for a wider historical context). While insufficiently discriminative in their own right, such logical inferences become vastly more powerful when supplied with alternative insights into the limitations of the psychological processes in question (for example, basic neurological insights into the properties of neurons and regional clusters of the brain).

The third, and perhaps final option for refining explanatory theories, is the independent discovery of design details (Lewens, 2002). In mechanical and electrical engineering, such insights may take the form of early blueprints, listing all available materials and tools, or learning what objectives a system was designed to implement (Dorst and Cross, 2001). In a manner wholly analogous to engineered design by humans, abundant evidence suggests that all organisms were designed (Dawkins, 2009), over a geological timescale, by a range of algorithmic evolutionary forces [see chapter 8 of Dennett (1995a), for further details].

Reliance upon details indicative of the design process is the central principle of the adaptationist approach, and is thus the heuristic core of evolutionary psychology (Buss, 2005). While embracing the adaptationist approach is not strictly necessary to gain some of the crucial benefits of the third aforementioned option (indeed any biological, medical, and developmental insights into the properties of the nervous-system provide powerful tools for use with the second and third), the adaptationist approach is designed to draw as much theory-guiding information as possible from the reciprocal relationships of form versus function. Stated simply, adaptationist heuristics regard what a mind *is* (structurally) as being intimately related to what a mind *does* (functionally), by in turn acknowledging that *how* a mind functions has been shaped by *why* it functions, in a Darwinian sense (Hodgson and Knudsen, 2008).

## Reverse-engineering and adaptationism

The paradigm of evolutionary psychology is primarily concerned with explanation, and this orientation has formed the basis of its conceptual incompatibility with the most prominent domains of differential psychology. By examining the explanatory methods employed in evolutionary research, the authors will demonstrate, by contrast, the explanatory short-cuts that have become entrenched in differential psychology, which keep differential researchers at odds not merely with adaptationists, but with theoretically robust psychology in general.

### The guidance of design

The adaptationist approach is the defining aspect of any work of evolutionary psychology (Sober, 2000; Buss, 2005). An adaptation (when used as a noun) is understood to be a feature or set of features of an organism, the apparent design or concerted complexity of which suggest that it is a product of natural selection, and thus represents a relational calibration of said organism to its ancestor's recurring environmental challenges (Tooby and Cosmides, 2005). At the heart of this paradigm is the suggestion that the species-typical behavioral and cognitive regularities of animals (of humans in particular), likely consist of, or are actively shaped by, adaptations.

Evolutionary psychologists focus on adaptations primarily for pragmatic, explanatory reasons. While all organisms are the products of natural selective forces (and artificial selection in domesticated species), not all features of organisms are adaptations. In the words of Tooby and Cosmides (2005, p. 25,26):

The cross-generationally recurrent design of an organism can be partitioned into (1) adaptations, which are present because they were selected for, (2) by-products of adaptations, which were not themselves targets of selection but are present because they are causally coupled to or produced by traits that were, and (3) noise, which was injected by the stochastic components of evolution.

For reasons of logical necessity, it is nearly impossible to use any positive criteria to confirm that some biological or psychological characteristic is either a by-product or phylogenetic noise. However, a feature can generally be identified as an adaptation when it shows contextual evidence of ‘good design’ with relation to the adaptive problem or problems it is hypothesized to address (Dennett, 1995a; Buss, 2005).

Functionally speaking, adaptations are relations between organism characteristics and the fitness demands which statistically favored those characteristics in their gene-pool (Dennett, 1995a; Sober, 2000; Dawkins, 2009). Thus, no trait can be accurately described as an adaptation in the absence of this feature-problem matching. For this reason, adaptationists approach complex features with the postulation of possible adaptations, moving on to the possibilities of by-products and noise when the evidence for adaptation is lacking or inadequate (Tooby and Cosmides, 2005). The tell-tale signs of biological design are the clues used by evolutionary psychologists to generate and refine theories about the probable structure and development of a psychological adaptation, utilizing the intrinsic relationships between the form and function of a well-designed system (Dennett, 1995a; Pinker, 1997, 2002; Tooby and Cosmides, 2005). Investigations of this sort are appropriately referred to as “reverse-engineering” (Buss, 2005), though it is worth noting that in seeking to gain insight into black box structures through inference from observable input-output contingencies, one could easily argue any psychologist employing explanatory theories is, by necessity, a reverse-engineer (Dennett, 1995b).

### Literal structures defined by function

Evolutionary theory regards the “mind” as a coordinated system of fitness-enhancing problem-solving apparatus. These hypothesized adaptations are specified to strictly consist of computational neurophysiological structures (Crawford, 2000; Cosmides and Tooby, 2001; Tooby and Cosmides, 2005). The existence, performance, and related properties of these adaptations are predicated upon the function they were selected for (Sappington, 1990; Keri, 2003). This focus on information-processes clearly lends itself to many process models in psychology, while many other targeted phenomena in psychology, such as intrinsic ‘traits’ (Church et al., 2006), internal representations (Fuhrman and Boroditsky, 2010), and qualitative mental states (Markus, 1998), can be understood as calibrated components, products, and observation-level descriptions of psychological processes (see Buss, 2005 for further detail). In contemporary evolutionary psychology, such structures are defined as psychological mechanisms, commonly further designated into processing “modules” (see Buss, 1995; Cosmides and Tooby, 2005, concerning the Massive Modularity Hypothesis).

This account of causally-integrated psychological mechanisms is vital to the conceptual lexicon of evolutionary psychology, and sets a clear yet inclusive standard for the compatible expression of any scientifically viable explanatory psychological construct (including those not thought to be adaptations). The viability of proposing such structures depends largely on evidence found in concerted phenotypic function. As such, the adaptationist approach also provides a unique means of bridging the gap between literal and non-literal construct-based theories, because any construct that is defined by its function is conceivable and testable as a literal, neurophysical psychological mechanism (Dennett, 1995b). Despite these evident benefits, it is precisely this concept of psychological mechanisms, and the detailed explanatory approach that such a conception demands, that is responsible for the much of incompatibility between the theories and approaches of evolutionary and differential psychology.

## Top-down explanations and descriptive constructs

There is perhaps no more fitting a characterization of differential psychology than as a field that endeavors to be descriptive. The methodologies and conceptual-tools of differential psychology are supremely well-adapted to the tasks of summarizing and extracting the statistical cores of behaviorally-recurrent trends in populations. With such immense statistical credentials, differential psychology is considered perhaps the greatest beneficiary of the above-mentioned quantitative imperative in behavioral science (Michell, 2003a). Indeed, researchers routinely seek to establish the real-world relevance of theory-based explanatory models (particularly concerning cognitive abilities and personality traits) through the use of differential descriptive constructs. It is telling that the opposite is only very scarcely the case.

The most prominent constructs in differential psychology, the general factor of intelligence “*g*” and the largely orthogonal personality trait dimensions of the Five Factor Model, were founded with few-or-no explanatory tasks in mind (Meehl, 1998; Lubinski, 2000), and have built their reputations instead on robust statistical properties and impressive correlations with life-outcomes. The “*g*” construct is an illustrative example, for contrary to common opinion, *g* is not an explicitly (linguistically) defined construct that is supported by a nexus of covarying statistical trends between many measures. Instead, “*g*” is simply a name given to a robust statistical nexus of covariation (Lubinski, 2000). Similarly, the orthogonal factor structures of the Five Factor Model of personality take precedence over any worded definition of the factors in question, in a sense making the definition of the factors intrinsically and permanently subject to interpretation (Cattell, 1996; McCrae and Costa, 1999; Grucza and Goldberg, 2007).

The esteem and popular use of such descriptive constructs has, however, led to their insertion into domains that do not match their original intentions or conceptual strengths. While differential descriptive constructs have proven their value through predictive correlations with achievement and outcome measures (Lubinski, 2000), in recent decades the literature has seen the rise and growing acceptance of individual differences papers which employ said descriptive constructs as proposed causative agents in simple explanatory theories (see Boag, 2011 for a detailed account). This form of explanation-description substitution produces a range of far-reaching conceptual problems, particularly with regard to circular reasoning and reification. As the following examples demonstrate, there are limited circumstances in natural science where empirical inquiry into antecedent causes cannot continue, and detailed description is embraced as a surrogate form of explanation. This explanatory approach is viable for only a minority of natural phenomena, and is intrinsically ill-suited for psychology and cognitive science.

### Limiting cases

When utilizing descriptive constructs in the role of causative agents, one is relying upon the assumption that reliable trends in observable behaviors are indicative of specific causal forces, be they agents or merely “laws” of expression (Boring, 1950). While this assumption is far from unheard of in some natural sciences, the subject matter of many scientific fields are not nearly as obfuscated by the black box limitations inherent to psychology. For two examples, consider the well-regarded fields of classical molar chemistry and moderate-scale Newtonian physics (Kitcher, 1985). These two fields have enviably few ambiguities in their subject matter, provided they are measured with sufficiently accurate instruments. Subsequently, both molar chemistry and Newtonian physics are founded upon reliable explanatory ‘laws’, such as Gay-Lussac's law or the Law of Universal Gravitation, all of which were discovered essentially atheoretically through the logical induction of observable trends. While these inquiries yielded theories, they did not require any assumed theoretical framework to undertake. In the terminology advocated by Cervone (1999, 2004, 2005), the explanatory method employed in these two examples, and subsequently misemployed when employing descriptive psychological constructs in explanatory roles, is referred to as *top-down* explanation (see also Kitcher, 1985; Salmon, 1989; Glennan, 2002).

Top-down explanation relies upon the induction of reliable, structural trends and distinctions, based purely on observational regularities. Of particular interest to psychologists, research programs that employ a top-down explanatory approach are directly compatible with population-level data, as inductions are best made statistically from a wide pool of nomothetic observations. In some sciences, such as chemistry and physics, sufficiently robust observational trends can be reliably assumed to correlate with fundamental causal forces, but such accounts are minority cases not to be confused with the wider sense of explanation, which relies upon giving accounts of causal antecedence (explored in-depth in Kitcher, 1985).

In the example of the Law of Universal Gravitation, Newton described in great detail the patterns of relative moment between bodies with mass, and ascribed the name gravity to the consistencies observed (Keesing, 1998). Thus in Newton's model, it is true that positing the force of gravity successfully explains the movement of objects with mass (within particular limits), but the phenomena of gravity itself remains merely described, and not explained at all. To this day, physicists struggle with competing theories in an effort to give a substantial antecedent-based explanation of gravitation and mass, but in Newton's era the viable limit of inquiry had been reached, and it was enough to say that the explanatory effort could end at a detailed description of the most fundamental accessible cause. Though such reasoning is inescapably circular, this description-explanation substitution was accepted due to the immense regularity of the patterns being observed, and because the phenomena in question are so fundamental and causally inscrutable, that the act of reification would not result in the premature dismissal of accounts of true causal antecedents. In psychology, however, this is far from the case.

### Misapplication

The most prominent contemporary example of descriptive constructs being invoked as top-down explanations of behaviors, are those centered on the Five Factor Model of personality (McCrae and Costa, 1994, 1997). The problems with attempting to use super-ordinate traits in this manner are two-fold: Firstly, psychological phenomena do not meet the conditions of simplicity and observational clarity required to employ an empirically coherent top-down analysis, as most relevant behaviors demand some interpretation or contextual inference to be studied (De Los Reyes and Kazdin, 2008). Human (and animal) behaviors are the result of many cumulative causal forces, whose patterns and configurations cannot in any way be directly induced from observable behavioral trends (Cervone, 2004, 2005). Second, these super-ordinate personality traits are proposed as explanations of the very behaviors that they are aggregated from. This represents internally-inconsistent circular reasoning, as a discrete phenomenon cannot be coherently understood to cause itself (Skinner, 1953; Hanson, 1958; Nozick, 1981; Bandura, 1999; Cervone, 2005; for a more complete treatment of the logical inconsistencies and reification errors in personality trait models, see Boag, 2011).

While the aforementioned conceptual problems are readily identified by those familiar with cases of circular reasoning, attention must also be drawn to the practical and methodological barrier between said constructs and explanatory theories in psychology. Although differential psychologists can and do utilize repeated-measures and other within-persons approaches, the majority of popular descriptive constructs are derived nomothetically, based upon between-persons patterns within sampled populations, and are thus befittingly labeled “difference variables” (Lubinski, 2000). Generally, these population-level variables are presumed to serve as indicators of some intrapersonal factor that determines an individual's contribution to the variation within a group, but as is pointed out by Borsboom and Dolan (2006), such assumptions cannot be embraced without empirical support. To simply presume equivalence between hypothetically related variables, when one exists on the individual-level and the other on the population-level, is conceptually unsound. These conceptual problems compound even further the more aggregated or abstracted a construct is from direct behavioral measurements. A clear example of this conceptual error can be found in the works of Kanazawa (2010a), which investigate “intelligence” as an adaptation for negotiating evolutionarily novel stimuli, while relying methodologically upon the general factor *g* (Kanazawa, 2006a,b,c, 2007; Lynn and Kanazawa, 2008; Kanazawa and Perina, 2009; Kanazawa and Reyniers, 2009; Kanazawa, 2010a,b). Kanazawa's theories presuppose the existence of a mechanism of general problem-solving, which is further assumed to correlate with population-level intelligence-differentials so closely that the *g* construct can be taken as its direct measure. As Borsboom and Dolan (2006) demonstrate, neither the probable existence of this mechanism, nor its presumed correlation to *g* have any substantive empirical or theoretical support. Conversely there are also a number of compelling reasons to believe that domain-general problem-solving mechanisms of the sort described cannot exist coherently in a computational framework (see Penke, 2011 for details). Kanazawa's use of *g* illustrates precisely the kinds of conceptual errors that arise when the untenable “top-down” explanatory approach native to differential psychology attempts direct integration with more robust theories, which rely upon a “bottom-up” approach to explanation.

## Bottom-up explanations and process models

In contrast to top-down explanatory methods, Cervone (1999, 2005) also speaks of their conceptual opposite, called simply “bottom-up” explanation. This is the form of explanation predominantly referenced throughout this paper, and is the approach required by adaptationism. Bottom-up explanations are comprised of either literally specified causal antecedents, or functionally-defined approximations of possible literal causal antecedents, hypothesized to underlie the phenomena of interest (Cervone, 2005). To varying degrees, all process models in psychology (specified at the level of an individual) are designed to employ a bottom-up explanatory approach, as they rely upon establishing the counterfactual causes of the phenomena in question (Edwards and Jaros, 1995). There are, however, two key conceptual limitations to the use of classical process models in seeking bottom-up explanations. The first issue concerns the relative completeness of a process account, while the second concerns the difficulty in addressing the first issue via the integration of multiple models.

### Incomplete and incompatible

Process models have been proposed to describe innumerable specific domains of cognition: the expression of innate temperaments (Richards, 1986; Eysenck, 1994; Mauer and Borkenau, 2007; Aron et al., 2010), the formation of attitudes (Tybout and Scott, 1983; Park et al., 2007), detail-extraction in perception (Marslen-Wilson and Warren, 1994; Vandenbroucke et al., 2009; Wascher and Beste, 2010), and in social learning processes in general (Bandura, 1986, 1989), to name only a few. Each of these examples demonstrates that strong theories of probable internal operations can (and must) be induced from a wide variety of formative and design-related clues. However, each theory is also fundamentally incomplete when considering the black box nature of the mind. In order to be reliably scrutinized via hypothesis-testing, a theory should account for at least some form of influence at all relevant stages of information-transformation between input stimuli and behavioral output. For example, a process account of reacting to a perceived stimuli should give some consideration to each stage of influence, from perception, to recognition, to motivation, to contemplation, and finally to expression, because variations at any of these levels would fundamentally change the observed input-output contingencies. While such a task may be impossible to achieve in exhaustive detail, and no theorist could be reasonably held to so lofty a standard, the more complete a theory's account of the causal sequence is, the lower the chances that some overlooked variable might skew or invalidate the results.

An intuitive solution to this issue would be to rely on existing process models of related psychological phenomena to supplement those points in a model where intervention would be meaningful. Unfortunately, the persistence of this problem can be largely attributed to issues of terminology, which present an obstacle to integration. Even those processes whose causal pathways of interest may appear mutually compatible are often kept separate by the incompatible referential terminologies of the fields from which they originate (Henriques, 2003). For example, Ho and Fung (2011) published a detailed process model of forgiveness, designed to account for some cultural influences on when and how forgiveness occurs and is displayed. By defining the process of forgiveness in terms of changes in affect and appraisal toward a transgressor, Ho and Fung adopted a functional approach well-suited to cross cultural comparisons, allowing for the simultaneous consideration of emotion, motivation, and other cognitions (for background on this approach, see Enright and Fitzgibbons, 2000). While this model does well by considering a wide variety of potential points of influence in the forgiveness process, some stages (deliberation and expression, in particular) are construed in such a manner as to leave their relationship to other published models vague. Rather than indicating how related models overlap with the stages described, or alternatively, justifying why the existing distinctions prevalent in the literature are inappropriate in this context, both interpretations appear potentially viable. For example, the model (p.79) defines a process of “dialectical thinking” as a major stage in forgiveness, but gives limited elaboration on what this consists of. From the descriptions, dialectical thinking appears to involve comprehension and attribution, cognitions that have also been addressed with cognitive process models in recent years (Rosset, 2008; Ali et al., 2011). Unfortunately, the authors neither acknowledge this potential overlap, nor explain why the terminology used is to be preferred. It seems that the possibility of integration was simply not considered, and that the distinctions employed in this model are idiomatic to the research task. Similarly, the forgiveness model accounts for cultural sources of variance in the emotion-negotiation and expression of forgiving sentiments, but not in a manner immediately compatible with prevailing process models of emotion-regulation (Ochsner and Gross, 2008; Thiruchselvam et al., 2011). It seems that with several basic changes to the defining terminology, this model of forgiveness could potentially be integrated with models of related phenomena, to yield testable predictions in far more substantive detail. Such conceptual clashes are par-for-the-course in psychology research, with only a minority of new theories showing explicit aspirations for wider integration (see Sheldon, 2011, as an example).

### Integration through adaptationism

The paradigm of evolutionary psychology offers a valuable potential solution: the standardization of referential language into the terminology of modern computational cognitive psychology (Cosmides and Tooby, 2000). An adaptationist theory must be either functionally-oriented toward behavioral outcomes, or hypothesize directly about literal psychological mechanisms. As such, employing evolutionary terminology ensures that effectively any process theory can be expressed in a manner highly compatible with many (and potentially all) other psychological mechanisms (Buss, 2005). Unlike other more abstract procedural concepts, adapted psychological mechanisms are conceptually primed to integrate on the basis of function (see Tooby and Cosmides, 2005 for further discussion). Beyond this, adaptationists can qualify meaningful predictions purely on the level of manifest behavior, because any well-designed adaptation must not interfere with the successful engagement of other mechanisms, except in explicit conditions of evolutionary mismatch (explained further in Cosmides and Tooby, 2001). In these two ways, the grounding theories of evolutionary psychology allow for potentially any process-based theory to be incorporated into more complete, conceptually sound, bottom-up theories. As such, adaptationist theories demonstrate a conceptual interplay between descriptive and explanatory tasks not commonly seen psychological science.

## The evolution of individual differences

As was explored in the preceding sections, the prevailing methods in differential psychology cater specifically to the scientific task of description, and are thus not only theoretically-impoverished with regard to explanation, but appear irreconcilable with more theoretically-robust approaches (Anderson, 2005; Muncer, 2011). These arguments are not to be taken as a general indictment of differential psychology, which remains a highly successful and instructive descriptive enterprise, but merely as a warning and reminder that top-down explanations are scientifically ill-suited to psychological phenomena.

The descriptive nomothetic data provided by prominent differential psychology constructs are commonly designed for highly generalized predictions of outcomes, rather than to provide details that disambiguate the mysteries of particular explanatory models (Lubinski, 2000). This explanatory neutrality represents the primary obstacle to researchers hoping to harness statistically powerful descriptions in aid of explanatory hypothesis-testing. Such researchers must struggle to interpret the meaning of quantitative differences that, as explored above, often do not easily map onto linguistic definitions (Cervone, 1999, 2004, 2005). If theorists hope to modify descriptive constructs to better inform causal explanations, population-level behavioral variations must be measured in a manner more indicative of the intrapersonal variables suspected to cause them (Borsboom and Dolan, 2006). That is to say, individual-differences measures must be adjusted so as to preserve (rather than control or mask) individual-level details that map onto the relevant features of explanatory theories. Without such considerations, any research paradigm seeking to bridge the gap between its specific hypotheses and the wider observations of differential psychology, must struggle in vain to match those elements in their explanatory theories thought to produce systemic variations, to the form said variation is expected to take on a generalized behavioral level.

Though some integration efforts have endured for decades (Buss, 1984, 2009), only in recent years have leading evolutionary psychologists embraced the task of modifying and expanding traditional adaptationist theories, in order to account not only for sources of random variation, but also variations preserved or arising from selective forces (Tooby and Cosmides, 1990; Confer et al., 2010; Buss and Hawley, 2011). The following section briefly details some recent expansions of evolutionary theory into areas once thought to be the exclusive purview of classical differential psychology.

### When selection maintains variation

Since the infancy of evolutionary psychology, Buss (1984, 1991, 1995, 2009) has explored the concept that a species may evolve a species-typical suite of adaptive interaction strategies (rather than a single “one size fits all” strategy), which are activated or deactivated developmentally as a means of calibrating an individual to the to the particular adaptive challenges of their lifetime (see also, Marsh and Boag, 2010). Despite the promise of this conception with regard to understanding personality psychology, this model presupposed a complex adapted system whose existence must be second-order to, and in principle shaped-by, the more basic selective influences thought to also produce systemic variation (Buss and Greiling, 1999). As such, the greatest advances over the past 10 years of variation-focused evolutionary psychology have comprised a range of sophisticated conceptual and empirical syntheses, aimed at exploring nuanced and often-overlooked Darwinian effects on the cognitive and dispositional properties of human individuals (Michalski and Shackelford, 2010). Speaking broadly, three largely distinct selective phenomena have been refined as viable sources of systemic individual differences in evolved psychology: First, that some dispositions and tendencies represent selectively-neutral or frequency-dependent fitness tradeoffs (as in the case of some personality traits). Second, that some abilities vary due their configural sensitivity to mutation-selection balance (as in the variables of human intelligence). Lastly, in accordance with Buss's founding insights, some psychological phenomena may vary as a function of niche-selecting mechanisms, be they cognitive or epigenetic. This final conception of variation remains largely in its infancy, and will not be discussed at length here (for a wide overview of the potential impact of this perspective on both addressing and redefining psychopathology, see Kennair, 2011).

With regards to fitness tradeoffs, early research (see Buss, 1995) investigated the influence that highly flexible, rapidly-changing environments, would likely have on the slow inter-generational process of trait-favoring selection in a population. Analysis suggests that some human ancestral environments may appear selectively-neutral by virtue of selective pressures either frequently changing, or being too contingent on intra-generational factors (see Belsky, 1999 for summary). This analysis was enriched by increasingly sophisticated tradeoff theories, hypothesizing that the fitness optima of highly variant traits are in fact their “moderate” as opposed to “high” levels, since extremes along many trait continuums are likely to confer maladaptive side-effects (see Keller and Miller, 2006; Nettle, 2006; Penke et al., 2007a,b; Ellis et al., 2009,for details). Building on these insights, theorists were able to account for the selective value of some seemingly disadvantageous, yet common, behavioral tendencies (such as those related to both competitive and altruistic social compulsions) via the inclusion of costly signaling theory (see Miller, 2007 for review) and life-history considerations (see Kaplan and Gangestad, 2005 for relevant discussion). These investigations gave rise to the study of frequency-dependent selection, wherein some variations are understood to be differentially effective based on the distribution of the same and other strategies employed by other members of the population (see Penke et al., 2007a,b for an introduction). With this wealth of insights, evolutionary psychologists now possess a sufficiently nuanced understanding of the selective pressures that likely underlie much of the systematic variation in personality and preference (Keller and Miller, 2006; Penke, 2011; Nettle, 2011).

In contrast, the traditional conception of adaptive optimization still appears to be relevant in studying the variations found in cognitive abilities and intelligence. Unlike variations in personality or preference, there appear to be very few tradeoffs or contingent circumstances that render higher levels of ability anything but an unambiguous enhancement of global fitness (Penke et al., 2007a). Fortunately, technological and analytical advances in population genetics have allowed the once-elusive concept mutation-selection balance to applied to the study of cognitive ability (Keller and Miller, 2006; Penke et al., 2007a,b). It has long been understood that the vast majority of natural mutations between the generations of a species tend to impair the collective functioning of their evolved adaptations. It is the ongoing role of natural selection to counteract this accretion of deleterious mutations by selecting against individuals with the greatest accumulation of impairments (individuals with a high effective “mutation-load”). The specific relevance of this phenomenon to cognitive abilities is due to the vulnerability of complex neurological adaptations to relatively small genetic impairments (Michalski and Shackelford, 2010). Since the configuration and optimization of complex psychological adaptations rely upon many structural and developmental provisions, the collective influence of many coordinated genes and expression-factors contribute to the formation of the delicate final product. Small changes to structural characteristics or enzyme efficiencies can thus result in measurable reductions in the calibrated efficiency of the overall mechanism (Keller and Miller, 2006). Thus, mutation-selection balance suggests that the majority of ordinal variation observed in the heritable characteristics of “intelligence,” are due largely to negative influences of mutation-loads not yet “filtered-out” by the omnipresent pressures of selection (Penke et al., 2007b), which in-turn partially explains some once-mysterious correlates of intelligence, including general health, vascular development, and body symmetry (Penke, 2011).

### Finding variation within mechanisms

The conceptual tools are now available to other researchers, including career differential psychologists, to begin bridging the divide between evolutionary intra-personal models and traditional individual differences methods. By engaging with explanatory process models, and building upon the elements of those models which permit of individual variations (both as heritable genetic biases or ontogenically calibrated strategies), new causally-relevant hypotheses can be tested with only minor modifications to existing psychometric techniques. Although the above-discussed modes of variation will likely be alien those without an evolutionary background, it is now well within the reach of differential psychologists to apply their methodological expertise, on both individual and population levels, to enriching even relatively simple process-based evolutionary theories.

The key to such efforts, however, is to embrace the lack of relevance most popular differential psychology constructs have to explanatory hypothesis-testing, and working to produce intermediary measurement tools and approaches that can bridge between the predicted variations within a process model, and what form said variation can be expected to take on an overt behavioral level. A strong example of this kind of research can be found in the social rank/dominance and social-exchange measures developed by Leybman and associates (Zuroff et al., 2010; Leybman et al., 2011a,b). Although the various incarnations of these measures resemble, both in presentation and in statistical verification, traditional differential psychometrics, fundamental design distinctions were taken directly from existing evolutionary process models of how humans negotiate reputation-sensitive social exchanges. Rather than creating and factor-analyzing a pool of items, with the goal of retroactively assigning descriptive titles to the factors that emerge, each element of the measures was intended to capture particular sources of intra-personal variation in the theorized psychological mechanisms, and their statistical validity was judged by how well response-patterns reflected this. Not only are these measures of dominance and social-exchange primed for testing hypotheses pertinent to the explanatory theory they are inspired by, their correlations with other descriptive constructs designed purely on the population-level can further inform an understanding of how intra-personal variations shape (and in the case of frequency-dependent selection, interact with) the overall diversity of the population (Leybman et al., 2011a).

In addition to providing more causally-relevant theoretical structures for the examination of variations already explored at the individual and population level, evolutionary-differential integration may also, on occasion, permit insightful conceptual revisions of some individual differences phenomena that have otherwise eluded explanation. For example, by expanding beyond the initial efforts of Rushton (1985, 2000, 2004), Figueredo and colleagues have developed a new approach to studying the General Factor of Personality (GFP), which utilizes life history strategy as the ultimate factor organizing the seemingly diffuse traits and behaviors observed (see Figueredo et al., 2005; Figueredo and Rushton, 2009). Beyond offering an account of the general organization of personality traits relating to social functioning, this approach has yielded a range of novel predictions concerning how ontogenic calibrations of life history strategy, such as degree of parental support, shape variation in GFP (van der Linden et al., 2012). Similar life history approaches have recently been applied to other domains of normative variation that have eluded simple explanation, including the clustering of several cognitive aptitudes and personality traits [as explored in Woodley et al. (2013)], and the human stress response system (see Del Giudice et al., 2011, for theoretical framing of the model, and Del Giudice et al., 2012, for promising empirical support). Each of these examples demonstrates a collection of psychological phenomena that had been successfully identified top-down as a reliable pattern of variation by differential psychologists, but which eluded explanation and a source of novel predictions in the absence of a functional account of evolved psychological mechanisms.

## Conclusion

In closing, this article has explored both the historical origins and contemporary impact of a perceived incompatibility between differential and evolutionary psychology, within the wider context of the unique challenges psychology faces as a science. The core of this incompatibility can be traced to confusions over, and a lack appreciation for, the distinct scientific tasks of description and explanation. Exclusive specialization in quantitative descriptive statistics has left differential psychology institutionally powerful, but theoretically-impoverished and conceptually isolated, with only limited means of applying its descriptive prowess to causal explanatory models. Evolutionary psychology has demonstrated a range of empirical and conceptual strengths that support its suitability as an integrating platform for functional cognitive and behavioral science. This strength has most recently manifested as a series of sophisticated and highly successful attempts to expand into the territories of differential psychology, thus establishing a range of innovative new means of describing and explaining the underlying causes of individual differences.

Researchers now have the foundations laid for them to develop new, theoretically-rich descriptive tools which can contribute directly to the hypothesis-testing of explanatory process models. Particularly when utilizing the heuristic tools of evolutionary psychology, even researchers inexperienced with adaptationism can work to bridge the conceptual gaps between our theories of functional, psychological mechanisms, and our accounts of tendencies and abilities in individuals and the population at large.

### Conflict of interest statement

The authors declare that the research was conducted in the absence of any commercial or financial relationships that could be construed as a potential conflict of interest.
